# Bullous lichen sclerosus: A rare differential diagnosis of a blistering genital dermatosis

**DOI:** 10.1016/j.jdcr.2025.10.041

**Published:** 2025-10-29

**Authors:** Sissy-Alina Waschkowski, Merit Kaeding, Thomas Schwarz, Stephan Weidinger, Katharina Boch

**Affiliations:** Departments of Dermatology, Venerology, and Allergology, University Hospital Schleswig-Holstein (UKSH), Kiel, Germany

**Keywords:** bullous dermatoses, bullous lichen sclerosus, chronic inflammatory dermatosis, genital dermatoses, lichen sclerosus

## Introduction

Bullous lichen sclerosus is a rare subtype of lichen sclerosus. To date, it was predominantly described in extragenital manifestations.^1^^,^^2^ In our case we highlight the diagnostic and treatment of a bullous lichen sclerosus localized on the glans penis of a 79-year-old patient. We were able to demonstrate the positive effect of short-term high-potent corticosteroids on the clinical outcome. Genital dermatoses often lead to an impaired quality of life. Therefore, a reliable and fast diagnosis and effective treatment options are essential. The most common genital dermatosis are infectious skin diseases (fungal, viral, and bacterial) or chronic inflammatory diseases. Blistering genital dermatoses are rare. Differential diagnoses of blistering conditions in the genital area may be autoimmune bullous dermatoses, bullous lichen planus and bullous lichen sclerosus.^1^^,^^3^^,^^4^

## Case report

**Fig 1:**
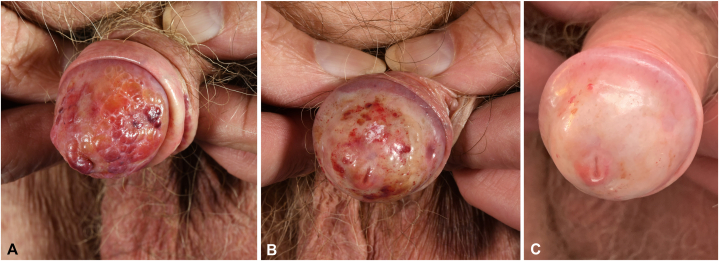
**A,** Clincial picture before treatment. **B,** Clinical picture after 4 weeks of topical treatment with clobetasol propionate ointment (twice daily). **C,** Clinical remisison at 16-week follow-up.

Our patient presented with a history of large, partly hemorrhagic blisters on the glans penis (Fig 1, *A*), persisting for over 2 years. The patient denied itching and pain. There was no indication of a correlation with a change in sexual partner or a traumatic event. The medical history did not reveal any further findings. Diagnostic swabs for viral and bacterial pathogens did not show an indication of an infectious disease. For further investigation, a punch biopsy was performed. Histopathologic findings showed a subepidermal blister containing erythrocytes, as well as an eosinophilic degeneration and homogenization of the subepidermal parts of the dermis. The findings supported the diagnosis of a bullous lichen sclerosus.

Thus, we initiated topical treatment with clobetasol propionate ointment (0.5 mg/g) twice daily for 4 weeks, which led to noticeable improvement of the condition and patients’ satisfaction (Fig 1, *B*). In the continuation of therapy clobetasol propionate was applied once daily for another 4 weeks, followed by 2 times per week with application of topical calcineurin inhibitors (pimecrolimus) on the remaining days. This led to a complete remission of the bullous condition after 6 weeks, with no side effects occurred. Residual findings such as hemorrhagic macules and hypopigmented areas remained up to 16 weeks after beginning of treatment (Fig 1, *C*).

## Discussion

The bullous variant of lichen sclerosus in the anogenital region is rare.^1^^,^^5^ The etiology remains unclear; genetic vulnerability, hormonal dysregulation, autoimmune, infectious, or traumatic influences are suggested triggers.^5^^,^^6^ Assumingly a vacuolar degeneration of the basal layer of the epidermis might lead to a loss of dermoepidermal junction, resulting in a subepidermal blistering.^6^^,^^7^ In addition, disruption of collagen support of the papillary dermis with increased fragility of the dermal capillaries is presumed to cause a hemorrhage within the lesions.^2^^,^^5^, ^6^, ^7^ There is also a debate about a cause through binding of antibodies against basement membrane zone components.^2^^,^^6^

Despite the unknown pathophysiology, this case demonstrates the successful therapy management of a rare genital bullous dermatosis, showing a complete remission after 16 weeks; contributing an important part to the knowledge of therapeutic management of bullous lichen sclerosus.^1^^,^^2^ To date, potent corticosteroid (eg, clobetasol propionate) is considered the most effective treatment for genital lichen sclerosus.^8^ Being the gold standard treatment, high-potency topical corticosteroids help to reduce inflammation and improve symptoms such as itching and pain.^9^ It was shown that high-potency topical corticosteroids are safe for long-term use in genital areas in adults as well as children.^9^^,^^10^ Further topical therapy options are calcineurin inhibitors combined with high-potent steroids for maintenance therapy, in line with our treatment regimen.^1^^,^^6^ Nonresponding to topical treatment, a systemic therapy attempt with oral corticosteroids, methotrexate, hydroxychloroquine, doxycycline, acitretin, or tofacitinib is warranted, given the limited amount of scientific data.^6^^,^^7^^,^^11^^,^^12^ Notably, these cases could not show evidence for the efficacy of a systemic therapy alone despite tofacitinib.^7^^,^^11^^,^^12^

## Conflicts of interest

None disclosed.
